# Prospectives for Gene Therapy of Retinal Degenerations

**DOI:** 10.2174/138920212801619214

**Published:** 2012-08

**Authors:** Gabriele Thumann

**Affiliations:** Universitätsaugenklinik / IZKF Aachen, RWTH Aachen, Pauwelsstr. 30, 52074 Aachen, Germany

**Keywords:** Adeno-associated viral vector, age-related macular degeneration, diabetic retinopathy, gene therapy, Leber congenital amaurosis, retinal degenerations, retinitis pigmentosa, Sleeping Beauty transposon, Stargardt disease.

## Abstract

Retinal degenerations encompass a large number of diseases in which the retina and associated retinal pigment epithelial (RPE) cells progressively degenerate leading to severe visual disorders or blindness. Retinal degenerations can be divided into two groups, a group in which the defect has been linked to a specific gene and a second group that has a complex etiology that includes environmental and genetic influences. The first group encompasses a number of relatively rare diseases with the most prevalent being Retinitis pigmentosa that affects approximately 1 million individuals worldwide. Attempts have been made to correct the defective gene by transfecting the appropriate cells with the wild-type gene and while these attempts have been successful in animal models, human gene therapy for these inherited retinal degenerations has only begun recently and the results are promising. To the second group belong glaucoma, age-related macular degeneration (AMD) and diabetic retinopathy (DR). These retinal degenerations have a genetic component since they occur more often in families with affected probands but they are also linked to environmental factors, specifically elevated intraocular pressure, age and high blood sugar levels respectively. The economic and medical impact of these three diseases can be assessed by the number of individuals affected; AMD affects over 30 million, DR over 40 million and glaucoma over 65 million individuals worldwide. The basic defect in these diseases appears to be the relative lack of a neurogenic environment; the neovascularization that often accompanies these diseases has suggested that a decrease in pigment epithelium-derived factor (PEDF), at least in part, may be responsible for the neurodegeneration since PEDF is not only an effective neurogenic and neuroprotective agent but also a potent inhibitor of neovascularization. In the last few years inhibitors of vascularization, especially antibodies against vascular endothelial cell growth factors (VEGF), have been used to prevent the neovascularization that accompanies AMD and DR resulting in the amelioration of vision in a significant number of patients. In animal models it has been shown that transfection of RPE cells with the gene for PEDF and other growth factors can prevent or slow degeneration. A limited number of studies in humans have also shown that transfection of RPE cells in vivo with the gene for PEDF is effective in preventing degeneration and restore vision. Most of these studies have used virally mediated gene delivery with all its accompanying side effects and have not been widely used. New techniques using non-viral protocols that allow efficient delivery and permanent integration of the transgene into the host cell genome offer novel opportunities for effective treatment of retinal degenerations.

## INTRODUCTION

The importance of “vision” can be assessed by the diversity of meanings of the word both in English and other languages as well as in the original Latin from which the word is derived. The meanings of “vision” range from the perception of light to divination of the future to an abstract and novel concept or theory to the reveries of the insane. The diversity of meanings attached to the word “vision” result from the fact that the physical act of “seeing” has allowed the acquisition of information that has led to the evolution of *Homo sapiens*, the intellectual man. We should note here that “vision” encompasses more than the simple activation of a light-sensitive pigment by photons and the consequent construal of a physical environment, which in its rudimentary form is found in organisms as simple as the unicellular *Euglenozoa* species.

“Vision” then encompasses not only the ability to perceive and construct images, but importantly the ability from a single image to “generalize”, retain the image in memory, assign a theoretical construct to the image, express and transmit such construct as information. Since the “visual” signals are processed by the visual cortex of the brain, not only is the construal of an image species specific but is individual specific. The individual uniqueness of image processing, interpretation and information acquisition is what has enabled humans to restructure the physical environment to increase survival of its species often to the detriment of other species.

Sight, the physical vision is a complex process in which the light-sensitive pigments, opsins, in the outer segment of photoreceptor cells absorbs a photon that converts *cis* retinal to *trans* retinal resulting in the activation of the G-protein transducin [1, 2], which activates cGMP-specific phosphodiesterase reducing intracellular cGMP. Reduced intracellular cGMP leads to closure of cyclic nucleotide-gated Na^+^-dependent ion channels, hyperpolarization of the photoreceptor cell membrane, closure of the Ca^+^-dependent ion channels, depolarization of the cell membrane and reduced release of glutamate. The electrical signal from the hyperpolarized photoreceptor membrane is transmitted via neurons to the visual cortex of the brain for processing into images and for decoding the images into information. In biological terms vision is the processing of light (photons) by the eye and brain. In its basic form, light colliding with the cornea is refracted, is apprized by the pupil, refracted and calibrated by the lens and vitreous, and acquired by the retina as an inverted image. The human retina (as well as the retina of many higher vertebrates) consists of specialized neurons, photoreceptors, that are able to transduce light into electrical signals; most vertebrates have two types of photoreceptors, rods and cones, which are morphologically and functionally different; rods are sensitive to dim light such that they can respond to a single photon, whereas cones function in bright light. In vertebrates cones are responsible for color vision and humans have three different types of cones that are sensitive to three different spectra and are designated as S, M and L to indicate that they are sensitive to short (peak sensitivity in the blue region of the spectrum), medium (peak sensitivity in the green region of the spectrum) and long (peak sensitivity in the red region of the spectrum) wavelengths. The electrical signals from the hyperpolarized photoreceptor membrane are detected by bipolar cells, processed and modified by horizontal and amacrine cell, assembled by retinal ganglion cells and conveyed to the visual cortex by the retinal ganglion cell axons, which are gathered to form the optic nerve. 

The complexity of the processes that allows sight and information acquisition from visible light presents innumerable possibilities for gene mutations, alteration in gene expression and alterations in regulatory interactions, which determine the precise spatial and temporal expression of each gene in the transcription “vision network”. Both gene mutations and alterations in gene processing can cause retinal degenerations. To date over 200 retinal-disease causing genes have been mapped and over 150 genes have been identified (http://www.sph.uth.tmc.edu/Retnet/sum-dis.htm#B-diseases last accessed September 2011).

Retinal degenerations present a wide variety of phenotypes with mild to severe vision loss and can be present at birth, develop during infancy or at any time during the life of the individual. Retinal degenerations are a progressive loss of retinal function caused by metabolic dysfunction and/or the progressive death of the cells of the retina. The effects on vision range from impaired vision, night blindness, loss of peripheral vision, loss of central vision to total loss of vision.

Retinal degenerations can be divided into two broad classes, hereditary retinal dystrophies and retinal degenerations. To the first class belong the monogenetic retinal dystrophies, which usually are inherited in a predictable pattern within families; hereditary retinal degeneration or dystrophies are relatively rare affecting approximately 1/3000 individuals in the Western world. The most prevalent hereditary retinal degenerations are retinitis pigmentosa (RP), Leber Congenital Amaurosis (LCA) and Stargardt Disease. 

To the second class belong age-related macular degeneration (AMD), glaucoma and diabetic retinopathy, which affect a large number of people worldwide. AMD affects over 30 million, diabetic retinopathy 40 million and glaucoma over 65 million individuals worldwide. The association of these diseases with age and diabetes represents a growing problem for the health care system since the proportion of the over 60 population is increasing dramatically in the world and with it there will be an increase in the number of people affected by these diseases. It is estimated that during the next 20 years the number of diabetics will reach over 350 million of which 75% will have some form of diabetic retinopathy within 20 years of the onset of diabetes. Likewise it is expected that the number of AMD and glaucoma cases will double within the next 10 years (ttp://www. who.int/blindness/Vision2020_report.pdf). Since these diseases are non-symptomatic until sight is affected, preventive treatments are usually not available and treatments of the disease once diagnosed are unsatisfactory or ineffective. 

For the well-being of the individual as well as for the burden that these diseases place on the health care systems, it is critical that treatments be discovered in the not-too-distant a future. Knowledge of the defective genes has engendered the hope that these diseases may be amenable to correction using gene therapy. However, even though the genes responsible for many retinal dystrophies have been known for a number of years [3-7] it has been only recently that gene therapy has shown some success in the treatment of LCA [8-12]. 

## HEREDITARY RETINAL DEGENERATIONS

### Retinitis Pigmentosa

Retinitis pigmentosa (RP) refers to a group of primary, chronic hereditary disorders in which photoreceptors and/or RPE cell abnormalities lead to progressive photoreceptor degeneration, which usually begins with defective dark adaptation (night blindness), progresses to abnormal color vision and eventually blindness [13-15]. The prevalence in the US and Europe is approximately 1/3,500 to 1/4,000 people; in China [16] the prevalence has been reported as 1/1000 and in India as1/930 people [17]. RP is characterized by multiple genetic inheritance patterns; in about 50% of cases RP occurs in patients without any known affected family members, in 20% of patients RP is inherited as autosomal dominant (adRP), in 20% of patients as autosomal recessive (arRP), and in 10 % of patients it is X-linked. RP is the result of mutations in anyone of over 100 genes and it can occur alone or in conjunction with other systemic disorders especially loss of hearing, which occurs in approximately 30% of patients [18]. http://www. ncbi.nlm.nih.gov/pubmed/17113430].

With the exception of the rhodopsin gene (*RHO*), which accounts for approximately 25% of adRP [4], the diverse genetic basis for RP, which is caused by a number of mutations in over 100 identified genes each mutation affecting one or more photoreceptor functions, makes gene therapy to correct the primary genetic defect unrealistic until gene therapy becomes so routine that can be individualized. Suppression and replacement therapies represent a treatment that circumvents the mutational heterogeneity in inherited disorders. Such an approach has been used in a mouse model of autosomal dominantly inherited rhodopsin-linked retinitis pigmentosa (RHO-adRP) [19]. Millington-Ward *et al.* using two adeno-associated virus (AAV) vectors co-administered shRNA to suppress the defective *RHO* gene and a replacement *RHO* gene to correct the defect in the transgenic *P347S* mouse model of RHO-adRP [20] observed significant functional (ERG) and structural beneficial effects, i.e. ONL retention and improved photoreceptor ultrastructure. 

An autosomal recessive form of RP is caused by a mutation in the phosphodiesterase-6 (PED6) of photoreceptors, which leads to an accumulation of cGMP and degeneration of photoreceptors. In the retina cGMP, produced by guanylate cyclase regulates ion channels; in the dark cGMP keeps the channel open allowing calcium entry into the cytoplasma while in the light PED6 hydrolyses cGMP closing the ion cGMP-gated ion channels (CNG). Absence or reduced levels of PED6 results in excess calcium in the cytoplasma, leading to photoreceptor degeneration [21]. In one of the first attempts at gene therapy in an animal model of retinitis pigmentosa with a mutation in PDE6, HIV vectors containing a gene encoding a hemagglutinin (HA)-tagged PDE6 were injected into the subretinal spaces of newborn *rd* mouse eyes. One to three rows of photoreceptor nuclei were observed in the eyes for at least 24 weeks post-injection, whereas no photoreceptors remained in the eyes of control animals at 6 weeks post-injection [22]. Delivery of wild type PED6 in the Pde6b^H620Q^ mouse model of RP using a lentiviral vector showed partial rescue of photoreceptors [23]. The addition of shRNA to the lentiviral vector to knockdown either guanylate cyclase (GUCY2E) or the cGMP-gated ion channel (CNGA1) did not increase photoreceptor rescue [24]. 

Another strategy for the treatment of RP is the rescue of photoreceptor cells by delivering to the photoreceptors neurotrophic factors; in fact, intravitreal delivery of ciliary neurotrophic factor (CNTF) has been shown to preserve cone cells and their function [25]. However, sustained delivery of a neurotrophic factor by intravitreal injection is limited by its logistics and short half-life. Another approach used is the delivery mediated by the adeno-associated virus (AAV) variant (ShH10), which is glial cell selective. Delivery of the gene for glial-derived neurotrophic factor (GDNF) mediated by AVV-ShH10 resulted in the secretion of high GDNF levels in treated retinas, leading to sustained functional rescue for over 5 months [26]. 

### Stargardt Disease

Autosomal recessive Stargardt disease (STGD1) was first illustrated and described by Stargardt in 1909 as a bilateral progressive atrophic macular dystrophy characterized by perimacular and peripheral "dirty grey-yellow spots" [27]. It is estimated that Stargardt disease, which usually affects children between the ages of 6 and 16 years, has a prevalence of one in 8,000 to 10,000 [28]. The first manifestations are loss of visual clarity, followed by gradual loss of central vision, progressive bilateral atrophy of the foveal retinal pigment epithelium (RPE) and photoreceptors, and often the macular region exhibits scattered deposits of yellow-orange lipofuscin deposits [29]. 

The disease is caused by mutations in the *ABCA4 (ABCR)* [30-32] gene which encodes a 210 kDa member of the family of ATP binding cassette proteins that is responsible for the transport of retinoid compounds across the outer segment disk membrane following the photoactivation of rhodopsin [33, 34]. The absence or insufficiency of ABCA4 protein results in the accumulation of all-trans-retinal, which react with ethanolamine, another component of outer segment membranes, to form the fluorophore A2E. A2E, the major hydrophobic component of lipofuscin, arises from the reaction of two molecules of all-trans-retinal with ethanolamine, both of which are molecular components of the photoreceptor outer segment membrane [35].

The one animal model for Stargardt disease is the abcr knockout mouse reported by Weng *et al.* [34]; these mice show increased accumulation of A2E in RPE cells and the delayed dark adaptation characteristic of Stargardt disease [36]. Subretinal delivery of the wild type ABCA4 gene using the equine infectious anemia virus (EIAV) vector resulted in high transduction efficiency of both rod and cone photoreceptors and reduction of A2E accumulation in RPE cells. [37].

### Leber Congenital Amaurosis

Leber congenital amaurosis (LCA) described by Theodor Leber in 1869 as a congenital form of retinitis pigmentosa [38] is one of the most severe retinal diseases. It is commonly inherited as autosomal recessive and it is caused by mutations in anyone of 14 genes (AIPL1, CABP4, CEP290, CRB1, CRX, GUCY2D, IQCB1, KCNJ13, LCA5, LRAT, RD3, RDH12, RPE65, RPGRIP1, SPATA7, TULP1); the rarer autosomal dominant form of LCA results from mutations in any of 3 genes (CRX, IMPDH1, OTX2). (http://www.sph.uth.tmc.edu/Retnet/sum-dis.htm#B- diseases). LCA is characterized by severely impaired visual acuity at birth or during the first 6 months of life, sensory nystagmus, poorly reactive pupils and severely diminished or non-detectable electroretinogram activity (ERG). LCA affects 1 in 35,000 newborn and represent 20% of all blind children [39, 40]. Since no successful treatment has been available for LCA, a number of investigators have endeavored to replace the defective gene and have had success in animal models of LCA [41-44]. One of the mutated genes that causes LCA is the RPE65 gene, which encodes for all-trans-retinyl-ester hydrolase, a 65 kDa enzyme that in RPE cells is critical for the production of 11-cis retinal. 11-cis-retinal is transported to the photoreceptors where it binds to apo-rhodopsin; the aporhodopsin-11-cis retinal complex reacts with a photon to produce a change in membrane potential, which generates a nerve signal that travels to the visual cortex for image formation and recognition. In RPE cells all-trans-retinyl-hydrolase is found in a soluble form that binds all-trans-retinol making it available for processing to all-trans-retinyl ester, which in turn is catalyzed by the membrane bound all-trans-retinyl-hydrolase to 11-*cis*-retinol.

Mutations in the RPE65 gene, which result in deficiency of all-trans-retinyl-hydrolase, account for about 6% of LCA cases in humans [45]. The natural occurrence of mutation in the RPE65 gene in dogs [46] and mice [47, 48] that mimics human LCA has made possible the development of gene therapy protocols for the successful treatment of these animals [49-56] as well as the application of these gene therapy protocols to the human disease. Using the AAV-2 vector to deliver the wild type RPE65 gene subretinally, the first gene therapy trials have been carried out by investigators at Children’s Hospital of Philadelphia and University of Naples, Italy (NCT00516477 – 3 patients) [57-59], at the Moorfields Eye Hospital and University College London, UK (NCT00643747 – 3 patients) [60], at the University of Pennsylvania, University of Florida and National Eye Institute, USA (NCT00749957 – 3 patients) [61, 62]; at the Scheie Eye Institute of the University of Pennsylvania in Philadelphia and at Shands at the University of Florida in Gainesville. (Investigational New Drug application BB-IND 12824; NIH Recombinant DNA Advisory Committee protocol 0410-677, 15 patients) [63]; the F M Kirby Center for Molecular Ophthalmology, Scheie Eye Institute, University of Pennsylvania (12 patients) [64]. 

The result of these trials have shown that delivery of the wild type RPE65 gene using the AAV serotype 2 vector is safe with no evidence of immunogenicity, inflammatory response or viral spread. Patients showed improvements in light sensitivity, nystagmus frequency and navigation around an obstacle. However, visual acuity improvement was less promising with only a limited number of patients demonstrating significant improvement.

## NON-HEREDITABLE RETINAL DEGENERATIONS

### Glaucoma

Second only to cataracts, glaucoma is the leading cause of blindness in the world. Glaucoma is a progressive optic neuropathy that progresses asymptomatically and is often associated with elevated intraocular pressure (IOP). Even though IOP is the most common diagnostic characteristic of glaucoma, it is not necessary or sufficient to cause glaucoma, since about 25% of glaucoma patients have normal IOP and many patients have elevated IOP but do not develop glaucoma. Glaucoma is classified as primary open angle glaucoma (POAG), primary acute closed angle glaucoma (ACG), and primary congenital glaucoma (PCG). It has been estimated that in 2010 glaucoma was responsible for over 8 million bilaterally blind people worldwide (POAG: 4.5 million, ACG: 3.5 million) and it is expected that by 2020 there will be over 11 million people bilaterally blind from glaucoma [65]. Whereas the prevalence of POAG and ACG appears to be similar in different ethnic populations, the prevalence of PCG varies widely. In Western countries the prevalence of PCG is 1:5,000-22,000, in the Middle East it is 1:2,500, in the Indian state of Andhra Pradesh it is 1:3,300; PCG accounts for approximately 4.2% of all childhood blindness [66] in Saudi Arabia and in the Roma population of Slovakia, PCG is the most common cause of childhood blindness [67, 68]. 

Except for PCG, which is associated with mutations in *CYP1B1*, the gene encoding cytochrome P450 1B1, and *LTBP2*, the gene encoding latent-transforming growth factor beta-binding protein 2, [69, 70] the genetic basis underlying POAG or ACG are mostly unknown. The most studied gene associated with POAG is MYOC, which encodes myocillin, a protein that is secreted into the aqueous and whose function is not known. Mutations in the MYOC gene are associated with approximately 4% of POAG. The function of myocillin is not known and decreased or elevated expression does not appear to cause glaucoma, rather it appears that mutations confer upon myocillin some unknown novel function(s) that may be causative for glaucoma [71-74]. Mutations in two other genes are associated with POAG, specifically mutations in the OPTN gene, which encodes optneurin a protein involved in regulation of membrane trafficking and cellular morphogenesis, and mutations in the WDR36 gene, which encodes a member of the WD repeat protein family involved in multi-protein complexes, signal transduction and gene regulation. Mutations in these 3 genes, MYOC, OPTN and WDR36, account for approximately 10% of all POAG cases.

Microenvironmental factors that may be responsible for the loss of retinal ganglion cells (RGC), optic nerve atrophy and vision loss include damage from elevated IOP, decreased nutrition from decreased blood flow, decreased levels of neuroprotective factors, oxidative stress, and glutamate toxicity [75-78]. Standard treatment for glaucoma relies on drugs to decrease IOP or surgery. Even though topical drugs to decrease IOP retard vision loss, these must be applied daily and in addition to non-compliance these drugs have unwanted side effects; surgery with implantation of devices to increase outflow is usually a last resort intervention. To provide long term protection to RGCs, neuroprotection has been advanced as a possible treatment for glaucoma [79]. Neuroprotection for RGCs has been approached by introducing into the vitreous stem cells [80-82], which produce neuroprotective factors as well as by delivering to the retina neuroprotective agents. Delivery of BDNF and PEDF using a lentiviral vector [83, 84] and electroporation to deliver CTNF [85] have been shown to protect RGCs from degeneration in animal models of glaucoma.

### Diabetic Retinopathy

Of the approximately 200 million people worldwide that suffer from diabetes approximately 40 million have diabetic retinopathy. These figures will increase dramatically over the next decades due to overall growth in population, increased life-expectancy, lifestyle that leads to lack of physical exercise, consumption of unhealthy and processed foods and obesity. Associated with diabetes is retinopathy, which in the United States affects 28.5% of diabetics over age 40 [86] and is the leading cause of blindness in people between the ages of 25 and 74 years.

The causes of retinopathy appear to be related to the persistent hyperglycemic environment, which results in oxidative stress, production of free radicals [87, 88], advanced glycemic end-products (AGE) [89, 90], pericyte damage and death [91-93], increased production of vascular growth factors [94-96] and an increase in vascular permeability [97, 98]. Microvascular lesions, such as microaneurysms, blood barrier dysfunction, and capillary dropout are key features of diabetic retinopathy [99-101]. Since the retinal vasculature supports the metabolic functions of retinal neurons and glial cells, these cells are damaged when the retinal vasculature is impaired. The damage can be prevented by controlling diabetes and blood sugar, although once occurred not all damage can be reversed; in fact, some alterations in gene expression engendered by diabetes are not reversed even by long-term control of blood sugar by insulin [102].

Since over time diabetic retinopathy will develop into proliferative diabetic retinopathy with proliferation of retinal vessels both into the retina and into the vitreous, treatment of diabetic retinopathy are being developed using inhibitors of VEGF to prevent blood vessel growth. Using adeno-associated virus serotype 2 (AAV2)-mediated delivery of genes encoding proteins that bind and inhibit the activity of VEGF, specifically sFLT01 [103] and chimeric forms of sFLT1 and sFLT02 [104], inhibits neovascularization in animal models of diabetic retinopathy. It has also been shown in animal models of diabetic retinopathy that delivery of genes using an adenoviral vector that express inhibitors of the urokinase plasminogen activator pathway, specifically amino-terminal fragment (ATF), and endostatin reduced retinal neovascularization [106]. Delivery of the vasoinhibitin gene mediated by AAV-2 has also been shown to inhibit VEGF and diabetes [107] induced neovascularization. 

### Age-Related Macular Degeneration (AMD)

Among the age-related diseases that affect vision, AMD is the most frequent cause of blindness in patients older than 60 years [107]. AMD is a chronic progressive disease that appears to result from poorly understood, age-associated alterations that include impaired phagocytosis by retinal pigment epithelial (RPE) cells, alteration in Bruch’s membrane, inflammatory reactions that lead to RPE cell degeneration followed by photoreceptor degradation, and eventually neural retinal ganglion cell degradation. AMD presents two distinct forms, a slow progressing non-neovascular (or avascular) atrophic form, which is characterized by RPE cell and photoreceptor degeneration, and a rapidly progressing, blinding neovascular form in which the ingrowth of new choroidal vessels through Bruch’s membrane damages the interface between Bruch’s membrane and RPE and between RPE and photoreceptors leading to RPE and photoreceptor degeneration. [108-110].

Treatments for avascular AMD are unavailable. Until 2006 treatments for vascular AMD consisted of laser treatment alone or coupled with intravitreal triamcinolone administration and/or choroidal neovascular membrane (CNV) removal. These treatments, however, did not result in significant visual improvement [111, 112]. Surgical removal of the CNV is accompanied by traumatic loss of the RPE cells while damaging the integrity of the Bruch’s membrane-RPE-photoreceptor complex and thus limiting visual prognosis in all types of subretinal surgery as shown in a meta-analysis by Binder’s group [113]. 

*In vivo*, the avascularity of the subretinal space appears to be dependent on the anti-angiogenic activity of PEDF, a factor that is expressed by many cells including RPE cells. In fact, it has been shown that expression of PEDF is highest in the developing fovea of midgestation human retinas and in the fovea of monkeys aged between fetal day 55 and 11 years at age [114]. In addition, in normal adult eyes PEDF concentration is 10-fold higher than VEGF in the macular region but not in the peripheral retina [115] strongly suggesting that PEDF is responsible for macular avascularity. Even though RPE cells secrete PEDF constitutively, the amount secreted in pathological conditions, such as AMD, is apparently not sufficient to control the neovascularization; in fact, PEDF levels have been found to be significantly lower in the vitreous of eyes from patients with proliferative diseases such as proliferative diabetic retinopathy (PDR) and AMD than in the vitreous of normal eyes [116, 117]. The demonstration that overexpression of VEGF and the decrease in PEDF expression in the retina are sufficient to elicit retinal and choroidal neovascularization [118, 119] led to the development of anti-angiogenic therapies for neovascular AMD. Since 2006, intravitreal administration of inhibitors of VEGF, particularly anti-VEGF monoclonal antibodies (Avastin^®^ or Lucentis^®^), have largely replaced surgical procedures for vascular AMD. The injection of anti-VEGF antibodies shows significant improvement in visual acuity with a gain of 3 or more lines in approximately 40% of patients and stabilization in 90% of patients [120, 121]; however the effect is limited in time by the short half-life, 10 days, of the antibodies *in vivo*, thus necessitating repetitive, often monthly injections to maintain the therapeutic effect. The repetitive injections carry substantial risks for the patient such as retinal detachment, endophthalmitis, cataract formation, ocular hypertension, submacular hemorrhage [122, 123] as well as the possibility that intravitreally injected VEGF inhibitors may diffuse systemically and cause thromboembolic events [124]. 

Since in the eye the physiological function of PEDF is the maintenance of a neuroprotective microenvironment to allow proper neuronal function, which includes the inhibition of abnormal retinal and choroidal blood vessel growth, increasing the level of PEDF would be the ideal therapy for the neovascularization that accompanies AMD and proliferative diabetic retinopathy. Although PEDF has important therapeutic capacities, its short half-life limits its clinical therapeutic use; in rats when applied subconjunctivally less than 1% of the protein reaches the choroid and the level decreases to less than 0.1% after 24 hours [27]. In an attempt to increase the levels of PEDF subretinally, RPE and IPE cells have been transplanted in animals and in AMD patients; even though in animal models of retinal degenerations the transplanted cells retard degenerations [126-130], in AMD patients the transplanted cells had no significant beneficial effects [131-135]. 

To inhibit the effect of the increased levels of VEGF a number of investigators have endeavored to deliver genes encoding inhibitors of VEGF into retinal tissues. In addition to PEDF, DNA constructs of VEGF receptors have been devised to express proteins that bind and inhibit the functional activities of VEGFs, i.e. sFLT01 [136] and Flt23K [137]. Intravenous injection of nanoparticles carrying Flt23K, a recombinant construct of VEGF-binding domains 2 and 3 of VEGFR-1/Flt-1 receptor coupled with the endoplasmic reticulum (ER) retention signaling sequence Lys-Asp-Glu-Leu (KDEL), was specific for the eye with neovascularization in a rat model of neovascularization and reduced the neovascular area compared to untreated animals [137]. Subretinal injection of adeno-associated virus encoding sFLT01 (AAV5.sFLT01) in Ccl2(-/-)/Cx3cr1(-/-) mice, a model for age-related macular degeneration (AMD) resulted in lower levels of A2E, the major component of lipofuscin, in better photoreceptor preservation and downregulation of retinal extracellular signal-regulated kinase (ERK) phosphorylation and inducible nitric oxide synthetase expression [138]. Subretinal deliver of sFLT01 and PEDF mediated by Sendai virus (SeV) vectors suppressed laser-induced choroidal neovascularization (CNV) in a mouse model; by 6 months histological examination showed that the retina of PEDF-treated animals was normal, whereas the retina of sFlt-1 treated animals showed photoreceptor degeneration associated with choroidal circulation defects [139]. A human trial to assess the effect of AAV2.sFLT01 is now recruiting patients (NCT01024998. Safety and Tolerability Study of AAV2-sFLT01 in Patients With Neovascular Age-Related Macular Degeneration (AMD) - http://clinicaltrials.gov).

In 2006 a report detailed the results of a phase I clinical trial in which 28 exudative AMD patients were treated with Ad-PEDF (adenoviral vector) delivered intraocularly. It was concluded that although 25% of patients experienced an inflammatory response, the therapy was safe and well tolerated [140]. No additional gene therapy trial or phase II study of this trial for AMD has been reported. 

## OPTIMIZATION OF GENE THERAPY FOR RETINAL DEGENERATIONS

Animal studies have shown that gene therapy for retinal degenerations is possible, and clinical translation has begun. However, for AMD the phase 1 clinical trial reported in 2006 [140] has not been followed and the trials for LCA-2 have had only limited success suggesting that improvements in gene therapy protocols, diagnosis and patient selection are needed for significant recovery of vision to occur in retinal degeneration patients. 

### Gene Therapy Protocols

Both in animal and in humans the protocols for delivering genes to retinal tissues have used adenoviral vectors or retroviral vectors. These vectors are efficient; however, they have shortcomings that would be best avoided. Adenoviral and adeno-associated vectors are epigenetic and since do not integrate into the host cell genome, expression of the transgene is not permanent and if the transgene function is to be maintained repeated delivery will be required with the accompanying risk of acute immune response [141] and possible dissemination in tissues far from the cell of interest [142]. Retroviral and HIV-based vectors integrate into the host genome but have a tendency to be mutagenic [143, 144]. Other gene-transfer methodologies, e.g. electroporation and lipofection, have been devised but are limited by low transfection efficiency, difficulty of gene targeting and cell damage [145]. The limitations of virally-mediated and of non-viral mediated methods of gene transfer can be overcome by using transposon mediated gene delivery. Transposons are discrete sequences of DNA that have the ability to move from one location and become integrated at another location of the genome within a single cell via a “cut and paste” mechanism called transposition. In nature transposons, in addition to the mobile DNA sequence, comprise a transposase gene flanked by terminal inverted repeats that contain the transposase binding sites. In a laboratory setting the gene of interest flanked by terminal repeats is carried by one plasmid and the transposase is carried by a second expression plasmid. The two plasmids are transfected into a host cell where the expressed transposase “cuts” the gene of interest from the donor plasmid and “pastes” it into a chromosomal site.

In vertebrates the great majority of transposons are inactive; however, a number of active transposons have been recovered and have been used for stable delivery of genes to cells in culture. Through the efforts of Professor Izsvak and colleagues in Berlin one transposon, *Sleeping Beauty*, not only has been reactivated [146, 147] but it has been reconstructed to be highly functional, efficient and safe; in fact, in 2009 the modified, “hyperactive” *Sleeping Beauty *[9] (SB100X) was accorded “Molecule of the Year” [148].* Sleeping Beauty *is a transposon belonging to the Tc1/mariner superfamily from teleost fishes [146], which can provide efficient stable gene transfer and sustained transgene expression in many cell types including primary cells [149-151] as well as in a number of animal models to correct a number of defects including Huntington’s disease. In a number of animal models the Sleeping Beauty transposon has been used to deliver genes to correct defects such as tyrosinemia, mucoplysaccaharidosis, and sickle cell anemia [152]. In the United States a clinical trial (NCT00968760) for B-Lymphoma using a modified Sleeping Beauty is now recruiting patients. *Sleeping Beauty and its modified forms *should be given consideration as a replacement for viral vectors whenever possible since it integrates genes exclusively into TA dinucleotides with specific predilection for TA dinucleotides that have distinct structural features in DNA sequences, regardless of primary DNA sequence [153, 154], integrates large inserts, does not integrate into transcriptional sequences, delivers and integrates transgenes stably and with high efficiency.

### Disease Diagnosis and Stage

Recovery of vision in retinal degenerations requires that the affected cells, RPE, photoreceptor and ganglion cells, be present and able to respond. However, the technology to diagnose whether a diseased cell can respond is not available. Optical Coherence Tomography (OCT), especially tridimensional OCT [155], is able to produce a morphological illustration of the retina and to identify RPE and photoreceptor cells; however, if RPE cells and or photoreceptors are irreversibly damaged, they may appear intact but may no longer respond or not respond fully to gene replacement, addition or subtraction. The absence of ERG’s and limited visual acuity recovery in LCA patients in which the defective RPE65 gene was replaced with the wild-type gene suggests that in addition to mutations in the RPE65 gene or perhaps because of such mutations, other cell functions have been compromised. New procedures are essential to assess whether RPE cells, photoreceptors and RGCs are still able to functionally respond to gene therapy.

If RPE cells and/or photoreceptors are degenerated or no longer responsive, strategies for cell replacement should be considered. A number of investigators have transplanted cells in animal models of retinal degeneration and shown that the transplanted cells prevent or retard retinal degenerations [127, 156, 159]. In humans cell transplantation has been attempted for the treatment of AMD. RPE cell transplantation is challenging since transplantation of homologous cells elicits immunological responses while transplantation of autologous RPE requires an involved surgical procedure with possible complications, such as proliferative vitreoretinopathy. Transplantation of autologous RPE cells as well as RPE-choroid explant in AMD patients has not shown promising functional results [160, 161]. To overcome these difficulties it has been proposed to use iris pigment epithelial (IPE) cells as a substitute for RPE cells, since autologous IPE cells can be easily harvested [162]. Extensive* in vitro *investigations have shown that RPE and IPE share many morphologic and functional similarities, including phagocytosis of outer segments and retinol metabolism [163-165]. In addition to PEDF, IPE cells synthesize BDNF, GDNF, NGF and neurotrophin-3, neuroprotective factors that may be necessary to maintain a healthy subretinal environment [166]. A number of animal studies have shown that IPE cells transplanted to the subretinal space are well tolerated [167, 168] and when transplanted to the subretinal space of Royal College of Surgeons (RCS) rats prevent the photoreceptor degeneration that characterizes the RCS rats [169]. Autologous IPE cells have also been transplanted into the subretinal space of end-stage AMD patients and even though there was stabilization of visual function, no significant improvement in visual acuity was observed [134, 135].

Upon analysis it was found that the IPE cells survived in the subretinal space but did not spread and colonize the areas where the RPE cells had degenerated, suggesting that the subretinal environment in AMD patients and possibly other retinal degenerative diseases is altered and not conducive to cell spreading and monolayer formation. Since to function properly cells transplanted to the subretinal space must form a monolayer and since cells transplanted in suspension do not appear to spread and form a monolayer, it will be essential to transplant cell monolayers attached to an artificial substratum. A number of substrata have been investigated as support materials specifically for transplantation of cells to the subretinal space [170-177], however cell monolayers have not yet been successfully implanted in an animal model of retinal degeneration.

An alternative to the transplantation of adult RPE or IPE cells is the transplantation of stem cells to replace degenerated RPE cells [178-181]. In a recent study mouse embryonic stem cells were differentiated *in vitro* into RPE precursor cells and then transplanted to the subretinal space of *Rpe65^rd12^/Rpe65^rd12^* mice, a model of retinitis pigmentosa. Of 123 mice transplanted with the stem cells differentiated *in vitro*, 76 mice developed tumors, subretinal masses or retinal detachments and only 12 showed an improvement in electroretinogram response [181]. This study is evidence that transplantation of stem cells to replace degenerated RPE cells is not feasible without additional studies and protocols that will prevent stem cells from forming tumors or replicate uncontrolled. Since IPE cells possess many of the characteristics and functionalities of RPE cells [164-166], are well tolerated into the subretinal space of human patients [134, 135], and autologous IPE cells can be easily obtained from a patient’s biopsy, autologous IPE cells should be the choice for RPE cell replacement in retinal degenerations. In this regard, recent studies have shown that IPE cells transfected with the gene for PEDF using the hyperactive Sleeping Beauty transposon system secrete significant amounts of recombinant PEDF, inhibit experimental corneal and choroidal neovascularization, promote neurogenesis of undifferentiated Y79 cells, and prevents photoreceptor degeneration in organ cultures of neural retina [182, 183].

Replacement of RPE by IPE cells or other cells will only be effective for vision recovery if the photoreceptors are not degenerated and are functionally able to respond to light. However if both RPE cells and photoreceptors are degenerated or irreversibly damaged the challenge to replace both and to have the cells assume the proper anatomical architecture is now only beginning to be explored. A number of investigators have shown that embryonic stem cells can be induced to differentiate into photoreceptor cells [184-186], which transplanted to the subretinal space of animal models of retinal degeneration can restore some visual functionality [187]. The numerous problems with transplantation of stem cells, including the requirement that the stem cells be differentiated *in vitro*, their immunogenicity, tumorigenicity, ethical issues, etc. present difficulties that would be best avoided. The difficulties associated with the use of embryonic stem cells can be overcome by the use of induced pluripotent stem (iPS) cells. iPS cells were originally derived from adult fibroblasts by the introduction and expression of four genes, namely OCT3/4, SOX2, KLF4 and c-MYC [188]. Since iPS cells derived by reprogramming adult cells using the oncogenes KLF4 and c-MYC, which when expressed can lead to the development of teratomas, a number of investigators have been successful at reprogramming adult cells into iPS cells using other gene sets [189, 190]. iPS cells have been shown capable of being directed to differentiate into a number of retinal precursor cells including RPE cells, cone and rod photoreceptors. and ganglion cells [191-194]. Transplantation of iPS-derived retinal precursor cells have been transplanted in animal models of retinal degeneration and shown to restore some visual functionality in animal models of retinal degenerations [187, 194]. Regaining vision by transplanting photoreceptor cells requires that RPE cells be present and functional; however, photoreceptors degenerate after RPE cells have degenerated and/or no longer function to support them; therefore transplantation of photoreceptors without transplantation of RPE cells will not restore vision in retinal degeneration patients. The acquisition of residual visual function in animal models of retinal degeneration by transplantation of photoreceptors very likely results from the secretion of neurogenic factors by functional RPE cells in the area adjacent to the transplanted photoreceptors. Acquisition of full functionality will require a functional monolayer of RPE cells. 

The ability to establish whether RPE cells and/or photoreceptors are degenerated in a patient by OCT should be the first to step in any protocol for treatment of retinal degenerations by cell transplantation. RPE cell replacement would be effective only if the photoreceptor layer is intact; if the photoreceptor layer is damaged, both RPE cell and photoreceptor replacement should be considered. However, the problem of replacing both RPE and photoreceptors will require substantial new research and novel approaches. In our laboratory we have begun to approach the problem of replacing both RPE cells and photoreceptors in AMD patients. We have developed protocols for the culture of autologous IPE cells transfected with the PEDF gene [194] cultured on ultrathin collagen type 1 membranes [173, 174] and the subretinal transplantation of these collagen-IPE cell constructs in the macular region when the photoreceptor layer is intact. Since transplantation of suspensions of RPE or IPE cells does not form a monolayer, it would be expected that transplantation of photoreceptor precursor cells would not integrate into the diseased retina of patients. Studies are, therefore, in progress to culture iPS-derived photoreceptor precursor cells on a collagen-IPE cell monolayer construct to re-engineer *in vitro* the essential architecture that mimics a Bruch’s membrane-RPE-photoreceptor complex. Transplantation of such a construct may integrate into the neural retina and establish the proper architectural connections to translate light into sight. 

## CONCLUSION

The eye is an ideal target for gene therapy treatments since it is confined, easily accessible and a number of retinal degenerative diseases are genetically well-characterized. Replacement of wild-type for the defective genes in animal models of retinal degenerations raised the hope that retinal degenerative diseases in humans would be amenable to gene therapy. Recently limited success has been attained for the treatment of LCA in patients with a defective RPE65 gene. Even though the specific gene defect may be known, the difficulty of treating the retinal degeneration patient is related to the degeneration of the RPE cells and/or photoreceptors regardless of whether the defect is in the RPE cells or photoreceptors. It is therefore essential that gene replacement therapy is instituted only in those patients in whom the RPE and photoreceptor layers are intact. Patients in which the RPE and/or photoreceptors are degenerated replacement gene therapy cannot restore vision. For these patients the expectation is that in the next few years cell replacement therapy, with or without genetic modification of the cells, will become a reality. The gene therapeutic innovations that are being introduced in ophthalmological medicine should open avenues that will be applicable to many other neurodegenerative diseases.
